# Bimetal Modulation Stabilizing a Metallic Heterostructure for Efficient Overall Water Splitting at Large Current Density

**DOI:** 10.1002/advs.202202750

**Published:** 2022-07-11

**Authors:** Tong Wu, Shumao Xu, Zhuang Zhang, Mengjia Luo, Ruiqi Wang, Yufeng Tang, Jiacheng Wang, Fuqiang Huang

**Affiliations:** ^1^ State Key Lab of High Performance Ceramics and Superfine Microstructure Shanghai Institute of Ceramics Chinese Academy of Sciences Shanghai 200050 China; ^2^ Center of Materials Science and Optoelectronics Engineering University of Chinese Academy of Sciences Beijing 100049 China; ^3^ State Key Laboratory of Rare Earth Materials Chemistry and Applications College of Chemistry and Molecular Engineering Peking University Beijing 100871 China

**Keywords:** bimetal modulation, interface engineering, large current density, metallic heterostructure, overall water splitting

## Abstract

Large current‐driven alkaline water splitting for large‐scale hydrogen production generally suffers from the sluggish charge transfer kinetics. Commercial noble‐metal catalysts are unstable in large‐current operation, while most non‐noble metal catalysts can only achieve high activity at low current densities <200 mA cm^−2^, far lower than industrially‐required current densities (>500 mA cm^−2^). Herein, a sulfide‐based metallic heterostructure is designed to meet the industrial demand by regulating the electronic structure of phase transition coupling with interfacial defects from Mo and Ni incorporation. The modulation of metallic Mo_2_S_3_ and in situ epitaxial growth of bifunctional Ni‐based catalyst to construct metallic heterostructure can facilitate the charge transfer for fast Volmer H and Heyrovsky H_2_ generation. The Mo_2_S_3_@NiMo_3_S_4_ electrolyzer requires an ultralow voltage of 1.672 V at a large current density of 1000 mA cm^−2^, with ≈100% retention over 100 h, outperforming the commercial RuO_2_||Pt/C, owing to the synergistic effect of the phase and interface electronic modulation. This work sheds light on the design of metallic heterostructure with an optimized interfacial electronic structure and abundant active sites for industrial water splitting.

## Graphical abstract

A metallic heterostructure Mo_2_S_3_@NiMo_3_S_4_ with enhanced electron transfer properties, fast reaction dynamics, and superior structural stability is designed and fabricated for large‐current alkaline water electrolysis. The constructed water electrolyzer operates at an ultralow cell voltage of 1.672 V@1000 mA cm^−2^ with ≈100% current density retention over 100 h, superior to the performance of commercial Pt/C║RuO_2_.

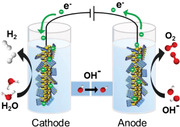

## 1 Introduction

**Figure 1:**
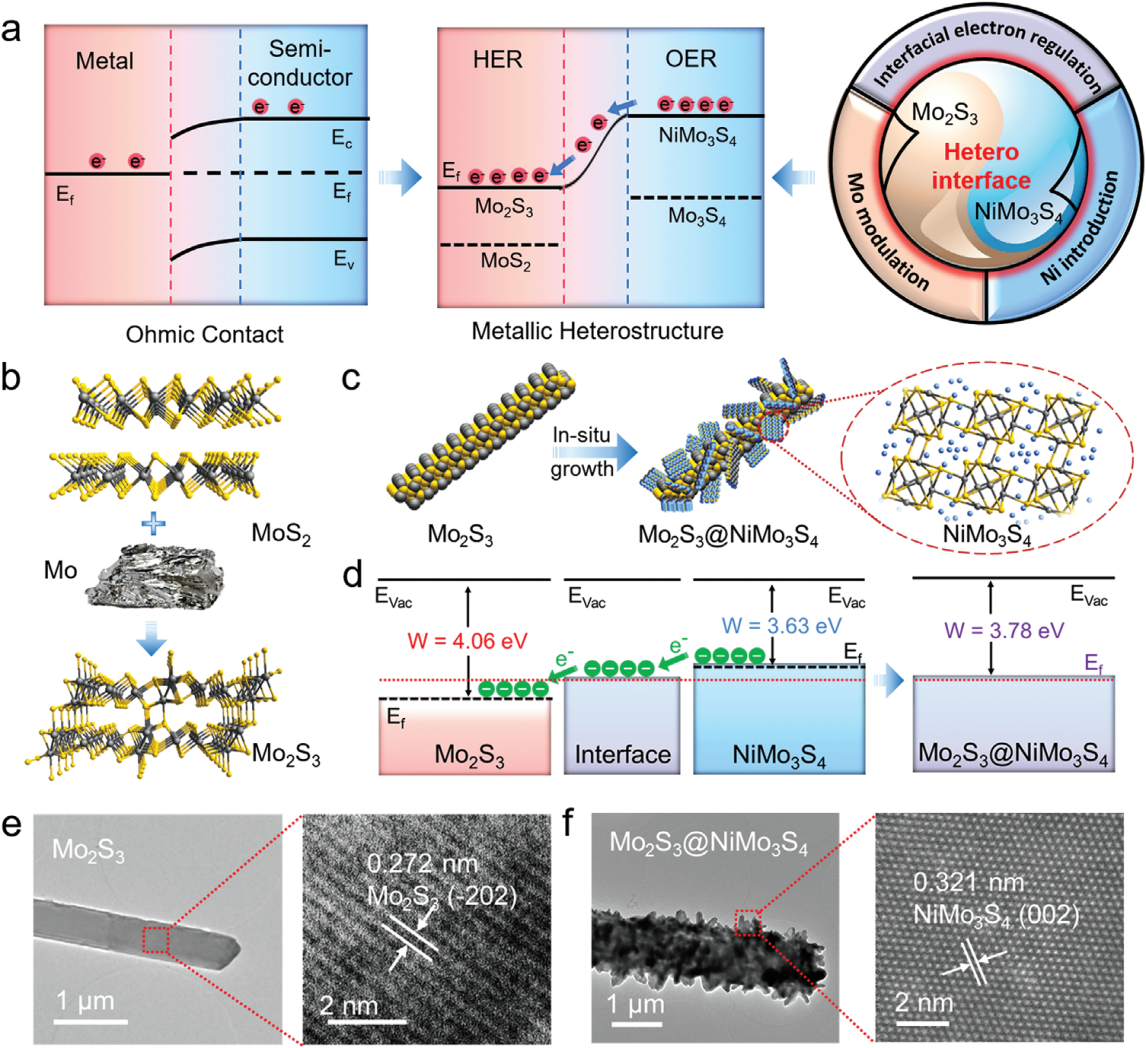
Design and characterizations for metallic heterostructure. a) Evolution of water‐splitting catalysts from typical heterostructure with ohmic barrier to the metallic heterostructure in this work with low Mott–Schottky barrier. b) Scheme of MoS_2_ converting to metallic Mo_2_S_3_. c) Schematic illustration of the epitaxial construction of Mo_2_S_3_@NiMo_3_S_4_. d) Energy band diagrams of Mo_2_S_3_ and NiMo_3_S_4_ (*E*_vac_ = vacuum energy, *E*_f_ = Fermi level, *W* = work function). e,f) TEM and aberration‐corrected TEM images of Mo_2_S_3_ (e) and Mo_2_S_3_@NiMo_3_S_4_ (f).

Electrochemical water splitting, including hydrogen evolution reaction (HER) and oxygen evolution reaction (OER), is a sustainable route for the continuous generation of hydrogen.^[^ ^1^, ^2^, ^3^, ^4^, ^5^, ^6^, ^7^, ^8^ ^]^ The commercial Pt/C and RuO_2_ have been regarded as typical catalysts for HER and OER, respectively, but the poor stability at large‐current densities and scarcity limit their wide application.^[^ ^3^, ^9^ ^]^ Despite aplenty reserves, most non‐noble metal electrocatalysts such as MoS_2_ and NiS_2_, have attracted extensive attention for alkaline water splitting, owing to their superior electrocatalytic performances at small current densities (<200 mA cm^−2^).^[^ ^10^, ^11^ ^]^ However, the limited bifunctional active sites and relatively poor stability result in the large overpotential and the degradation of electrochemical performance at large current densities over 500 mA cm^−2^,^[^ ^12^ ^]^ seriously restricting their application in the industrial high‐output water splitting. Typically, the industrially‐used Raney Ni electrocatalysts operating at 500 mA cm^−2^ for overall water splitting required a cell voltage of 2.4 V,^[^ ^13^ ^]^ which largely exceeds the thermodynamic potential of 1.23 V. To date, the overall water splitting catalyzed by NiMoO*_x_* @NiMoS*_x_*,^[^ ^14^ ^]^ FeP@Ni_2_P^[^ ^15^ ^]^ and NiMoN@NiFeN^[^ ^16^ ^]^ still required the cell voltages as high as 1.75, 1.73 and 1.72 V, respectively, at 500 mA cm^−2^. Although tremendous progress by regulation of phase structure, defects, interface, and active sites has been developed to boost activities of electrocatalysts for robust water splitting,^[^ ^17^, ^18^, ^19^, ^20^, ^21^, ^22^, ^23^, ^24^, ^25^, ^26^, ^27^ ^]^ traditional electronic regulation to construct a semiconductor heterostructure toward overall water splitting is difficult to realize for durable and fast Volmer H* and Heyrovsky H_2_ generation for large‐current operation, owing to the instinct of relatively low electronic conductivity and high ohmic contact resistance^[^ ^11^, ^28^, ^29^ ^]^ (**Figure** **1**a).

To address the above issues for robust overall water splitting, we propose a metallic heterostructure with the following advantages to satisfy the industrial demands: i) superior electrical conductivity for efficient electron transport; ii) sufficient reactive active sites for fast hydroxyl capture at large‐current densities; iii) excellent structural stability in alkaline medium at large‐current densities. In particular, the metallic sulfide support for the epitaxial growth of metallic heterostructure could be prepared by phase modulation of MoS_2_ with additional Mo implantation. The Mo modulation with the presence of additional Mo–Mo bonding with coordinately unsaturated centers can not only provide active sites for H* and OH* adsorption with reduced water dissociation barrier, that is the kinetically limited step in alkaline medium,^[^ ^30^, ^31^, ^32^ ^]^ but accelerate the desorption kinetics of H* and OH* with tunable local coordination structures in the alkaline medium.^[^ ^33^ ^]^ Furthermore, we construct the metallic heterostructure with enhanced bifunctional activities by incorporating Ni into the sulfide framework. On the one hand, the introduction of Ni with asymmetric 3*d* orbitals could recombine with the vacant 4*d* orbitals of Mo atoms in the sulfide framework, which can improve the interfacial electron transfer, weaken the proton adsorption and provide abundant active sites^[^ ^34^, ^35^ ^]^ (Figure 1a). Moreover, the strong interaction of Mo with Ni in constructed metallic heterostructure is promising for conquering the challenge of OH‐induced oxidation in alkaline electrolytes.^[^ ^36^ ^]^ On the other hand, the introduction of Ni into the Mo_3_S_4_ framework with electronic modulation and structural rearrangement could weaken the proton adsorption and provide abundant active sites to enhance Heyrovsky H_2_ generation.^[^ ^37^, ^38^, ^39^, ^40^, ^41^ ^]^

In this work, a metal sulfide, Mo_2_S_3_ with metallic conductivity, transformed from typical MoS_2_ with additional Mo–Mo bonding is utilized as the metallic support to in situ epitaxially grow NiMo_3_S_4_, for the construction of the metallic heterostructured Mo_2_S_3_@NiMo_3_S_4_, which has metallic conductivity and abundant active sites enabling the water electrolysis at large‐current densities. Meanwhile, the in situ epitaxial growth of NiMo_3_S_4_ nanosheets on Mo_2_S_3_ nanorods with low interfacial resistance and enhanced interfacial charge transfer properties are favorable for the fast OER dynamics. The rationally designed Mo_2_S_3_@NiMo_3_S_4_ metallic heterostructure with coupled 2D nanosheets and 1D nanorods shows superior electrocatalytic performance with small overpotentials of 173, 256 and 390 mV for OER, and 32, 124 and 174 mV for HER at 10, 100 and 1000 mA cm^−2^. When it was integrated into a symmetric two‐electrode electrolyzer, water electrolysis required only 1.639 and 1.672 V at industrial current densities of 500 and 1000 mA cm^−2^ with outstanding durability (over 100 h), which is among the best non‐noble electrocatalysts for large‐current water splitting.

## 2 Results and Discussion

### 2.1 Design and Characterizations for Metallic Heterostructure

Figure 1b shows the schematic illustration of semiconductor MoS_2_ converting to metallic Mo_2_S_3_ by introducing excessive Mo in MoS_2_ with the appearance of Mo–Mo bonds.^[^ ^42^ ^]^ The transformation of typical semiconductor MoS_2_ to metallic Mo_2_S_3_ can be revealed by the following Equation (1):

(1) $$3{\mathrm{MoS}}_{2}+\mathrm{Mo}\overset{\Delta }{\to }2{\mathrm{Mo}}_{2}S_{3}$$

Compared with MoS_2_, the conductivity of Mo_2_S_3_ was greatly improved due to the formation of Mo–Mo coordinated vertical zigzag chains. The metallic heterostructured Mo_2_S_3_@NiMo_3_S_4_ was constructed by the in situ generation of NiMo_3_S_4_ nanosheets on the Mo_2_S_3_ nanorods via a facile hydrothermal method (Figure 1c). The introduction of metallic Mo_2_S_3_ with a high Fermi level as support was conducive to interfacial charge transfer. Meanwhile, Mo_2_S_3_ possessing shorter Mo—Mo bonds than pure Mo metal could generate delocalized electrons and electronic states for tuning the adsorption/desorption behavior of H* and OH* with enhanced HER dynamics. The energy band diagrams of Mo_2_S_3_, NiMo_3_S_4_ and Mo_2_S_3_@NiMo_3_S_4_ revealed the favorable electron transfer from NiMo_3_S_4_ to Mo_2_S_3_ through the formation of heterointerface (Figure 1d). The ultraviolet photoelectron spectroscopy (UPS) spectra of a secondary edge region of Mo_2_S_3_, NiMo_3_S_4_, and Mo_2_S_3_@NiMo_3_S_4_ were further measured to characterize the interfacial charge polarization (Figure S1, Supporting Information). The work function (*W*) corresponding to the energy difference between Fermi level (*E*_f_) and vacuum level was calculated according to the equation of *W* = *hν* − *E*_cutoff_, where *hν* is the incident photon energy (21.22 eV) and *E*_cutoff_ is the normalized secondary electron cutoff.^[^ ^43^, ^44^ ^]^ The work functions of Mo_2_S_3_, NiMo_3_S_4_ and Mo_2_S_3_@NiMo_3_S_4_ are 4.06, 3.63 and 3.78 eV, respectively, which indicates the electron transfer from NiMo_3_S_4_ to Mo_2_S_3_ through heterointerface, consistent with the energy band diagrams.

The scanning electron microscopy (SEM) and transition electron microscope (TEM) images exhibited that the as‐synthesized Mo_2_S_3_ was nanorods with a smooth surface (Figure 1e and Figure S2, Supporting Information), and the characteristic lattice fringes of 0.272 nm matched the (−202) plane of Mo_2_S_3_. The NiMo_3_S_4_ nanosheets were observed to be well distributed onto Mo_2_S_3_ nanorods (Figure 1f and Figures S3–S5, Supporting Information). The spherical aberration‐corrected TEM image with atomic resolution showed the lattice fringes with interplanar spacings of 0.321 nm, attributable to the (002) plane of NiMo_3_S_4_. The corresponding elemental mappings displayed the uniform distribution of Ni, Mo, and S (Figure S6, Supporting Information).

**Figure 2:**
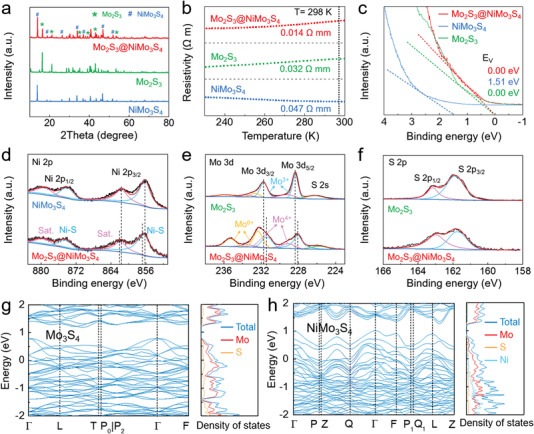
Structural characterizations of metallic heterostructure. a–c) XRD patterns (a), temperature dependence of the resistivity (b), and UPS spectra for low energy onset regions (c) of Mo_2_S_3_, NiMo_3_S_4_ and Mo_2_S_3_@NiMo_3_S_4_. d) Ni 2p XPS spectra of NiMo_3_S_4_ and Mo_2_S_3_@NiMo_3_S_4_. e,f) Mo 3d and S 2p XPS spectra of Mo_2_S_3_ and Mo_2_S_3_@NiMo_3_S_4_. g,h) Band structure and density of states of Mo_3_S_4_ (g) and NiMo_3_S_4_ (h).

The crystal structure of the as‐synthesized samples was determined by the X‐ray diffraction (XRD) patterns. The representative peaks attributed to the Mo_2_S_3_ phase (JCPDS No. 40–0972) confirmed the successful formation of Mo_2_S_3_ without other impurities (**Figure** **2**a). After epitaxial growth by hydrothermal reaction, new peaks attributed to trigonal NiMo_3_S_4_ (JCPDS No. 30–0847) were observed, revealing the successful formation of Mo_2_S_3_@NiMo_3_S_4_ composites. The temperature dependent resistivity was performed to investigate the electrical conductivities (Figure 2b). The electrical resistivity of Mo_2_S_3_@NiMo_3_S_4_ was 0.014 Ω mm at 298 K, which was ahead of that of Mo_2_S_3_ (0.032 Ω mm) and NiMo_3_S_4_ (0.047 Ω mm), illustrating its excellent electrical conductivity. Remarkably, the electrical resistivities of Mo_2_S_3_ and Mo_2_S_3_@NiMo_3_S_4_ decreased with reduced temperature, indicating that both of them are metallic.^[^ ^45^, ^46^ ^]^ Further characterization by UPS spectra at low energy onset regions of Mo_2_S_3_, NiMo_3_S_4_, and Mo_2_S_3_@NiMo_3_S_4_ confirmed the metallic properties of Mo_2_S_3_ and Mo_2_S_3_@NiMo_3_S_4_ (Figure 2c).

The electronic properties and valence states of the as‐synthesized Mo_2_S_3_, NiMo_3_S_4_, and Mo_2_S_3_@NiMo_3_S_4_ were analyzed by X‐ray photoelectron spectroscopy (XPS). The high‐resolution Ni 2p XPS spectra of NiMo_3_S_4_ showed that the peaks centered at 856.1 and 873.9 eV with two Ni satellite peaks could be assigned to characteristic Ni^2+^ spin‐orbit signals (Figure 2d). Compared with NiMo_3_S_4_, these peaks of Mo_2_S_3_@NiMo_3_S_4_ shifted slightly toward higher binding energy, indicating the partial oxidation of Ni^2+^. The peaks in Mo 3d XPS spectra of Mo_2_S_3_@NiMo_3_S_4_ located at 228.1/231.5 and 232.7/235.8 eV could be indexed to Mo^3+^ and Mo^6+^ components, respectively^[^ ^47^ ^]^ (Figure 2e), while the main peaks in S 2p XPS spectra at 161.6/163.4 eV were related to the S 2p_3/2_ and 2p_1/2_ orbitals of S^2−^ in Mo_2_S_3_ (Figure 2f). Therefore, these results implied that the selective epitaxial growth of sulfide species can change the electronic structures and coordination environments of the metal active sites with the asymmetric electron distribution and a reduced electronic loss ability in an electrocatalytic reaction, which is conducive to the enhanced catalytic activity.^[^ ^48^ ^]^ DFT calculation revealed the reduced bandgap upon introduction of Ni into the typical semi‐conductor Mo_3_S_4_ crystal to form NiMo_3_S_4_, and the intensity of partial density of states of NiMo_3_S_4_ was higher than that of Mo_3_S_4_ at the Fermi level, suggesting the favorable electron mobility^[^ ^49^ ^]^ of the NiMo_3_S_4_ catalysts (Figure 2g,h).

**Figure 3:**
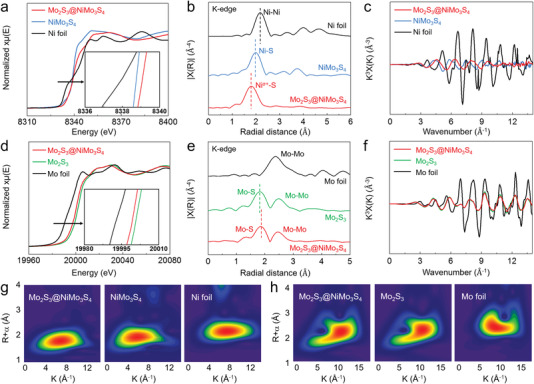
Electronic structures of metallic heterostructure. a–c) Ni K‐edge XANES spectra (a), R‐space EXAFS spectra (b) and corresponding oscillations (c) of Ni foil, NiMo_3_S_4_ and Mo_2_S_3_@NiMo_3_S_4_. d–f) Mo K‐edge XANES spectra (d), R‐space EXAFS spectra (e) and corresponding oscillations (f) of Mo foil, Mo_2_S_3_ and Mo_2_S_3_@NiMo_3_S_4_. g,h) Wavelet transforms for the k3‐weighted Ni K‐edge EXAFS (g), and Mo K‐edge EXAFS (h).

To determine the valance states and local coordination structures of Mo_2_S_3_@NiMo_3_S_4_, the X‐ray absorption near‐edge structure (XANES) and extended X‐ray absorption fine structure (EXAFS) were performed. The pre‐edge feature of Ni in the Ni K‐edge XANES spectra of Mo_2_S_3_@NiMo_3_S_4_ shifted slightly to a higher energy than that of NiMo_3_S_4_ (**Figure** **3**a), confirming the oxidation of Ni,^[^ ^50^ ^]^ consistent with the XPS results. The k3‐weighted EXAFS spectra in Figure 3b showed that the peaks of Mo_2_S_3_@NiMo_3_S_4_ (1.9 Å) and NiMo_3_S_4_ (2.0 Å) shift significantly to the shorter radial distance, compared with the Ni foil (2.3 Å, Ni–Ni coordination), confirming the presence of Ni–S coordination.^[^ ^51^ ^]^ The decrease in oscillation intensity oscillation curves of Ni K‐edge for Mo_2_S_3_@NiMo_3_S_4_ of Mo_2_S_3_@NiMo_3_S_4_ revealed an increase in disorder due to the existence of two crystal phases (Figure 3c).

The Mo K‐edge XANES spectra displayed that the pre‐edge feature of Mo in Mo_2_S_3_@NiMo_3_S_4_ shifted slightly to lower energy than that of Mo_2_S_3_ (Figure 3d), indicating the electron transfer from NiMo_3_S_4_ to Mo_2_S_3_. The R‐space shows the coordination peaks at ≈1.90 and ≈2.50 Å, which could be assigned to the Mo–S and Mo–Mo peaks (Figure 3e). Compared with the Mo–S peak of Mo_2_S_3_ (1.85 Å), the characteristic Mo–S peak of Mo_2_S_3_@NiMo_3_S_4_ shifted to a longer radial distance (1.90 Å), suggesting the electron transfer from Ni to Mo. The oscillation curves of Mo K‐edge for Mo_2_S_3_@NiMo_3_S_4_ and Mo_2_S_3_ also revealed the decreased oscillation intensity of Mo_2_S_3_@NiMo_3_S_4_ (Figure 3f). The wavelet transformed (WT) Ni K‐edge EXAFS oscillation was further performed to reveal the atomic dispersion.^[^ ^52^, ^53^, ^54^, ^55^, ^56^ ^]^ Compared with the maximum intensity of Ni foil (Ni–Ni contribution) at ≈2.3 Å, the maximum intensities of Mo_2_S_3_@NiMo_3_S_4_ and NiMo_3_S_4_ at ≈1.9 and ≈2.0 Å can be attributed to the Ni–S contributions (Figure 3g). Meanwhile, the maximum intensities of Mo_2_S_3_@NiMo_3_S_4_ and Mo_2_S_3_ at ≈2.30 and ≈2.35 Å, smaller than that of Mo foil (2.45 Å) suggested the presence of the Mo–S contributions (Figure 3h).

### 2.2 Electrocatalytic OER and HER Performance

**Figure 4:**
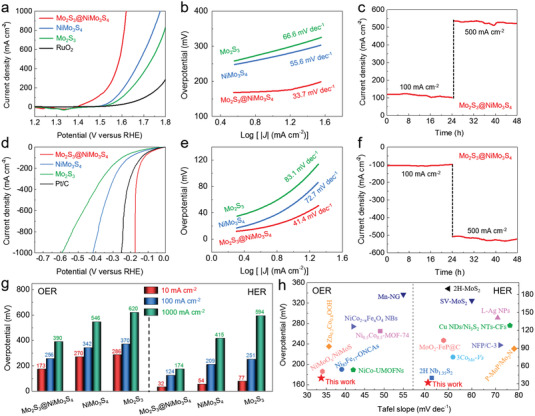
Electrocatalytic OER and HER performance. a,b) OER polarization curves (a) and corresponding Tafel plots (b) of Mo_2_S_3_, NiMo_3_S_4_, and Mo_2_S_3_@NiMo_3_S_4_ in 1 m KOH. c) OER chronoamperometry curves of Mo_2_S_3_@NiMo_3_S_4_ at 100 and 500 mA cm^−2^ in 1 m KOH. d,e) HER polarization curves (d) and corresponding Tafel plots (e) of Mo_2_S_3_, NiMo_3_S_4_, and Mo_2_S_3_@NiMo_3_S_4_ in 1 m KOH. f) HER chronoamperometry curves of Mo_2_S_3_@NiMo_3_S_4_ at 100 and 500 mA cm^−2^ in 1 m KOH. g) The overpotentials required at 10, 100 and 1000 mA cm^−2^ of Mo_2_S_3_, NiMo_3_S_4_ and Mo_2_S_3_@NiMo_3_S_4_ for OER and HER. h) Comparisons of kinetics (Tafel slope) and activities (the overpotential at 10 mA cm^−2^) for OER and HER.

The electrocatalytic performances of Mo_2_S_3_, NiMo_3_S_4_, and Mo_2_S_3_@NiMo_3_S_4_ supported on nickel foam were evaluated by the linear scan voltammogram (LSV) in O_2_‐saturated 1.0 m KOH solution at room temperature (**Figure** **4**a). The Mo_2_S_3_@NiMo_3_S_4_ required the overpotentials (*η*) of 173, 256 and 390 mV to deliver 10, 100 and 1000 mA cm^−2^, respectively, which were considerably lower than that of Mo_2_S_3_ (286, 370, and 620 mV), NiMo_3_S_4_ (270, 342, and 546 mV), and commercial RuO_2_ (306, 475, and >800 mV). It might be induced by the weak proton adsorption capacity of Mo_2_S_3_@NiMo_3_S_4_. To investigate the OER kinetic mechanism, the Tafel plots of Mo_2_S_3_, NiMo_3_S_4_ and Mo_2_S_3_@NiMo_3_S_4_ were performed (Figure 4b). Compared with Mo_2_S_3_ (66.6 mV dec^−1^) and NiMo_3_S_4_ (55.6 mV dec^−1^), Mo_2_S_3_@NiMo_3_S_4_ achieved the lowest Tafel slope of 33.7 mV dec^−1^, indicating the fast OER kinetics. The chronoamperometry curves of Mo_2_S_3_@NiMo_3_S_4_ were tested to evaluate the durability. No obvious change at the current densities of 100 and 500 mA cm^−2^ over 48 h exhibited superior long‐term stability (Figure 4c).

To investigate the electron transfer during OER, the in situ electrochemical impedance spectra (EIS) of Mo_2_S_3_, NiMo_3_S_4_, and Mo_2_S_3_@NiMo_3_S_4_ at different potentials were measured (Figure S7, Supporting Information). When the applied voltage increased to 1.35 V (versus RHE), an obvious mutation occurred in the Nyquist plots, indicating that OER began to occur. The lower charge transfer resistance (*R*_ct_) of Mo_2_S_3_@NiMo_3_S_4_ than that of Mo_2_S_3_ and NiMo_3_S_4_ demonstrated an enhanced charge transfer property of metallic heterostructure. In addition, the electrochemical double‐layer capacitance (*C*_dl_) was calculated through cyclic voltammetry (CV) curves to estimate the permittivity and the electrochemically active surface area (ECSA)^[^ ^57^, ^58^, ^59^ ^]^ (Figures S8 and S9, Supporting Information). Compared with Mo_2_S_3_ (17.1 mF cm^−2^) and NiMo_3_S_4_ (20.3 mF cm^−2^), Mo_2_S_3_@NiMo_3_S_4_ has a *C*_dl_ of 24.8 mF cm^−2^, even superior to commercial RuO_2_ (Figures S10 and S11, Supporting Information), suggesting the superior kinetics of metallic heterostructure with exposed abundant active sites for large‐current operation.

The HER performance of Mo_2_S_3_@NiMo_3_S_4_ was obtained by LSV in N_2_‐saturated 1.0 m KOH solution at room temperature, using Mo_2_S_3_, NiMo_3_S_4_, and commercial Pt/C as the reference samples (Figure 4d). The Mo_2_S_3_@NiMo_3_S_4_ displayed a very low overpotential of 32, 124 and 174 mV at 10, 100 and 1000 mA cm^−2^, which was superior to Mo_2_S_3_ (77, 251, and 594 mV), NiMo_3_S_4_ (54, 209, and 415 mV) and commercial Pt/C (41, 140, and 254 mV). A small Tafel slope, 41.4 mV dec^−1^ of Mo_2_S_3_@NiMo_3_S_4_ was achieved in comparison with Mo_2_S_3_ (83.1 mV dec^−1^) and NiMo_3_S_4_ (72.7 mV dec^−1^), demonstrating the rapid HER kinetics (Figure 4e). Moreover, the Mo_2_S_3_@NiMo_3_S_4_ can maintain the HER activities at 100 and 500 mA cm^−2^ after stability measurements over 48 h, showing outstanding durability (Figure 4f).

The EIS spectra of Mo_2_S_3_, NiMo_3_S_4_, and Mo_2_S_3_@NiMo_3_S_4_ were also collected during HER at different overpotentials (Figure S12, Supporting Information). The *R*_ct_ of Mo_2_S_3_@NiMo_3_S_4_ was much lower than those of Mo_2_S_3_ and NiMo_3_S_4_. A large *C*_dl_, 36.9 mF cm^−2^ was achieved for Mo_2_S_3_@NiMo_3_S_4_, much higher than that of Mo_2_S_3_ (13.9 mF cm^−2^), NiMo_3_S_4_ (18.4 mF cm^−2^) and commercial Pt/C (24.6 mF cm^−2^) (Figures S13–S16, Supporting Information). Accordingly, the Mo_2_S_3_@NiMo_3_S_4_ had a highly electrocatalytic performance for both OER and HER in alkaline media, and can even be operated in a large‐current density of 1000 mA cm^−2^ with a low overpotential of 390 mV for OER and 174 mV for HER (Figure 4g), much lower than those of Mo_2_S_3_ (620 and 594 mV) and NiMo_3_S_4_ (546 and 415 mV). The fabricated Mo_2_S_3_@NiMo_3_S_4_ catalyst had comprehensive advantages in low overpotential for both OER (Table S1, Supporting Information) and HER (Table S2, Supporting Information), which was superior to commercial RuO_2_, Pt/C, and the most reported recent electrocatalysts (Figure 4h). Based on the above results, Mo_2_S_3_@NiMo_3_S_4_ showed the remarkably high OER and HER electrocatalytic performance in alkaline media, which was mainly attributed to the interfacial electronic engineering in metallic heterostructure with high activity and conductivity.

### 2.3 Overall Water Splitting with Enhanced Reaction Dynamics

**Figure 5:**
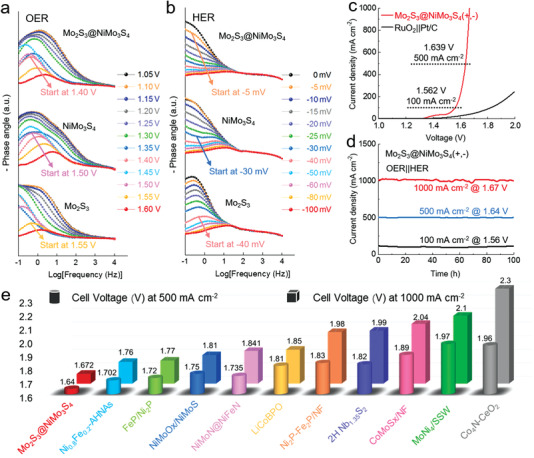
The in situ EIS measurements and overall water splitting performance. a,b) Bode phase plots of the in situ electrochemical impedance spectra of Mo_2_S_3_, NiMo_3_S_4_, and Mo_2_S_3_@NiMo_3_S_4_ for OER (a) and HER (b). c) Polarization curves by a two‐electrode system of Mo_2_S_3_@NiMo_3_S_4_||Mo_2_S_3_@NiMo_3_S_4_ and RuO_2_||Pt/C. d) Chronoamperometric tests of Mo_2_S_3_@NiMo_3_S_4_||Mo_2_S_3_@NiMo_3_S_4_ at 1.56, 1.64, and 1.67 V in 1 m KOH. e) Comparisons of the cell voltages at 500 and 1000 mA cm^−2^ of Mo_2_S_3_@NiMo_3_S_4_ with reported bifunctional electrocatalysts.

To investigate the reaction dynamics, the Bode phase plots of the in situ EIS measurements were performed on Mo_2_S_3_, NiMo_3_S_4_, and Mo_2_S_3_@NiMo_3_S_4_. An apparent transition peak was observed on Mo_2_S_3_@NiMo_3_S_4_ at the potential of 1.40 V (versus RHE) for OER, which was significantly lower than that of Mo_2_S_3_ (1.55 V) and NiMo_3_S_4_ (1.50 V), suggesting the faster reaction dynamics (**Figure** **5**a). Meanwhile, compared with Mo_2_S_3_ (40 mV) and NiMo_3_S_4_ (30 mV), the Mo_2_S_3_@NiMo_3_S_4_ exhibited an earlier transition peak at the overpotential of 5 mV for HER, demonstrating the rapid electron transfer (Figure 5b). A symmetric electrolyzer of Mo_2_S_3_@NiMo_3_S_4_ was further assembled for overall water splitting in 1.0 m KOH solution. The Mo_2_S_3_@NiMo_3_S_4_ (+,−) couple exhibits a low cell voltage of 1.56, 1.64 and 1.67 V at 100, 500 and 1000 mA cm^−2^, respectively, far superior to that of commercial electrocatalyst RuO_2_||Pt/C (Figure 5c). The Mo_2_S_3_@NiMo_3_S_4_ (+,−) electrolyzer can retain outstanding overall water splitting performance with no noticeable degradation at current densities of 100, 500 and 1000 mA cm^−2^ over 100 h with the low cell voltage (Figure 5d), which is currently one of the best bifunctional electrolyzers among non‐noble materials^[^ ^11^, ^60^, ^61^, ^62^, ^63^, ^64^, ^65^, ^66^, ^67^, ^68^, ^69^, ^70^ ^]^ (Figure 5e and Table S3, Supporting Information).

To explore the effect of metallic heterostructure on the durability of electrocatalytic reaction in alkaline medium, we fabricated the NiS_2_ nanoparticles for comparison. During the OER process, NiOOH would also be formed on the surface of NiS_2_ catalyst. However, after several cycles, most of the pristine NiS_2_ particles would convert into NiOOH, and S was dissolved into the electrolyte (Figure S17, Supporting Information). The XRD patterns of Mo_2_S_3_@NiMo_3_S_4_ before and after OER show no significant change, indicating that the structure is stable during water splitting (Figure S18, Supporting Information). The construction of metallic heterostructure with a strong interaction of Mo with highly active Ni atoms could improve the structural stability for large‐current operations. Moreover, the introduction of the metallic Mo_2_S_3_ could not only increase the electrical conductivity but also provide abundant defective sites at the heterointerface, thus promoting HER activities. Meanwhile, the strong interaction between Ni and Mo in the heterointerface of the catalyst was capable of increasing the interfacial electronic densities and reducing the protons‐adsorption energy, favorable for the enhanced catalytic activities.

### 2.4 Active Sites for Oxygen Evolution

**Figure 6:**
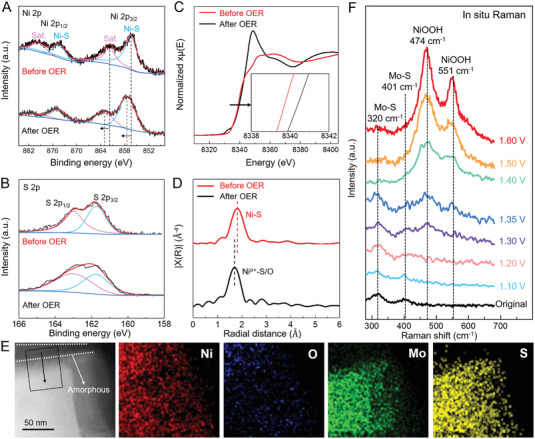
Active sites for oxygen evolution. a,b) High‐resolution XPS of Ni 2p (a) and S 2p (b) spectra of Mo_2_S_3_@NiMo_3_S_4_ before and after OER. c,d) Ni K‐edge XANES spectra (c) and R‐space EXAFS spectra (d) of Mo_2_S_3_@NiMo_3_S_4_ before and after OER. e) STEM and corresponding elemental mapping images of the Mo_2_S_3_@NiMo_3_S_4_ after OER. f) In situ Raman spectra of the Mo_2_S_3_@NiMo_3_S_4_ at various OER potentials.

To further investigate the catalytic active sites of the Mo_2_S_3_@NiMo_3_S_4_, the characterizations of structure, surface information, valance states and local coordination were studied after OER. The high‐resolution Ni 2p XPS spectrum displays two peaks located at 856.3 (Ni–S) and 862.0 eV (Sat.), positively shifting to higher energy attributable to the formation of Ni^3+^ oxyhydroxides species (**Figure** **6**a). The S 2p XPS results showed no distinct change as shown in Figure 6b. Furthermore, the Ni K‐edge XANES spectrum of Mo_2_S_3_@NiMo_3_S_4_ exhibited the distinct shift of Ni pre‐edge to higher energy after OER, demonstrating the oxidation of Ni to a higher valence state (Figure 6c). Compared to the original Mo_2_S_3_@NiMo_3_S_4_ (1.9 Å), the main peak of Mo_2_S_3_@NiMo_3_S_4_ after OER in k3‐weighted EXAFS spectra shifts significantly to the shorter radial distance (1.7 Å) attributed to the existence of Ni–O coordination, suggesting the formation of Ni oxyhydroxides (Figure 6d).

The STEM image, energy dispersive X‐ray spectroscopy (EDS) line scan, and corresponding elemental mappings of the Mo_2_S_3_@NiMo_3_S_4_ after OER displayed that an amorphous layer of Ni oxyhydroxides was formed on the surface (Figure 6e and Figure S19, Supporting Information). To further confirm the structure transformation during water splitting, the in situ Raman measurement was carried out to clarify the real‐time evolution of the Mo_2_S_3_@NiMo_3_S_4_ catalyst during OER (Figure 6f). From 0 to 1.2 V, Raman spectra showed only two peaks at 320 and 401 cm^−1^, which belong to Mo–S. Another two new peaks at 474 and 551 cm^−1^ at 1.3 V, corresponding to Ni^3+^–O bending peak and Ni^3+^–O stretching peak, respectively revealed the transformation into NiOOH from 1.3 V. When the potential exceeded 1.4 V, the peak intensities of NiOOH increased significantly. Combined with the above results, a thin amorphous layer of NiOOH was verified to form on the surface of Mo_2_S_3_@NiMo_3_S_4_ during OER, which facilitates the overall water splitting.

## 3 Conclusion

In summary, we developed a metallic heterostructure, Mo_2_S_3_@NiMo_3_S_4_ with enhanced electron transfer properties, fast reaction dynamics and superior structural stability to boost the water electrolysis at large‐current densities by synergistically modulating phase structure via Mo and Ni incorporation. The constructed heterostructured Mo_2_S_3_@NiMo_3_S_4_ achieves an extraordinarily low overpotential of 173 mV for OER and 32 mV for HER at 10 mA cm^−2^. The cell voltage of Mo_2_S_3_@NiMo_3_S_4_ couples’ electrolyzer at a current density of 1000 mA cm^−2^ is 1.672 V, and the peak current density remains stable after chronoamperometric test over 100 h, which is among the best non‐noble metal‐based overall water splitting electrocatalysts as yet. Based on the XAFS, in situ Raman, and in situ Bode phase plots, the excellent catalytic performance can be attributed to the following aspects: i) the in situ epitaxial growth of NiMo_3_S_4_ nanosheets on Mo_2_S_3_ nanorods provides abundant active sites; and ii) the metallic conductivity of Mo_2_S_3_@NiMo_3_S_4_ improves electron transport and reaction dynamics. Therefore, the designed Mo_2_S_3_@NiMo_3_S_4_ with much‐boosted electrocatalytic performance and excellent durability is a promising electrocatalyst for overall water splitting even at large‐current densities, which opens up new opportunities for developing efficient and stable electrocatalysts to meet the industrial demand in future.

## 4 Experimental Section

### Synthesis of Mo_2_S_3_

The Mo_2_S_3_ was synthesized via the high temperature molten salt method. Mo powder, S powder and NaCl powder were mixed in the molar ratio of 2:3:100, then they were ground thoroughly in a mortar. The mixed powders were pressed into a pellet and sealed in an evacuated quartz tube, which was transferred into a muffle furnace and annealed at 950 °C for 2000 min with a heating rate of 5 °C min^−1^. After calcination, the quartz tube was quickly taken out and cooled in cold water. The sample was collected and soaked in water to remove NaCl to obtain the Mo_2_S_3_ sample. The as‐obtained samples were dried in a vacuum oven at room temperature.

### Synthesis of Mo_2_S_3_@NiMo_3_S_4_

100 mg of Ni(NO_3_)_2_·6H_2_O and 50 mg of Mo_2_S_3_ were added to 20 mL of distilled water in a beaker under magnetic stirring. After ≈5 min, 2 mL of oleylamine and 10 mL of ethanol were quickly added and the stirring continued for 0.5 h to produce a homogeneous solution, which was then transferred into a 50 mL Teflon‐lined autoclave. The autoclave was sealed and maintained at 180 °C for 15 h in a convection oven and then naturally cooled to room temperature. The sample was collected and washed with cyclohexane, distilled water and ethanol to remove organics, ions and possible remnants, and dried in a vacuum oven at room temperature. The freshly obtained sample and 270 mg of (NH_4_)_2_MoS_4_ were added to 20 mL of DMF followed by stirring for 15 min under ambient conditions. Then, 0.1 mL of N_2_H_4_·H_2_O was added to the suspension. After stirring for another 15 min to dissolve completely, the mixed solution was transferred to a 50 mL Teflon‐lined autoclave. The autoclave was sealed and maintained at 200 °C for 15 h in a convection oven and then cooled to room temperature naturally. The resulting black sample was collected and washed with ethanol four times and dried in a vacuum oven at room temperature.

### Material Characterization

Scanning electron microscope (SEM) images were obtained by JEOL JSM6510. TEM images were obtained by JEOL JEM‐ARM300F. The spherical aberration‐corrected atomic resolution TEM images were obtained by Hitachi‐HF5000. X‐ray diffraction (XRD) characterization was carried out by Bruker D8 advanced diffractometer operating with Cu K*α* radiation. The electrical properties measured in the temperature range of 230–300 K were performed using a Quantum Design physical properties measurement system (PPMS). XPS measurements were run on a Thermo Scientific Escalab 250Xi. UPS measurements were performed on a Thermo Scientific Escalab 250Xi. The X‐ray absorption spectra (XAS) including XANES and EXAFS of the sample were collected at the Beamline of TPS44A1 in National Synchrotron Radiation Research Center (NSRRC), Taiwan. Raman spectra were obtained using a thermal dispersive spectrometer with laser excitation at 633 nm.

### Electrochemical Experiments

The electrochemical experiments were carried out in a three‐electrode cell using a CHI760E electrochemical workstation at room temperature. The ink was prepared by dispersing 5 mg of the catalyst and 100 µL of 5 wt% Nafion solution in ethanol (900 µL) by ultrasonication for 30 min to form a homogeneous dispersion. Then, 100 µL of the dispersion was drop‐casted onto a nickel foam (NF), leading to a catalyst loading of 2.5 mg cm^−2^. During the electrochemical measurements, carbon rod and Hg/HgO electrode were used as the counter and reference electrodes, respectively. The potentials reported in this work were calibrated to the RHE. In 1.0 m KOH, *E* (RHE) = *E* (Hg/HgO) + 0.059 × pH + 0.098 V. The overpotential (*η*) was calculated according to the formula: *η* = *E* (RHE) − 1.23 V. The KOH electrolyte was bubbled with N_2_ and O_2_ for 0.5 h before HER and OER test. Polarization curves were recorded by LSV at a scan rate of 5 mV s^−1^ in the range of 1.0 to 1.8 V versus RHE for OER, 0.0 to −0.7 V versus RHE for HER. To estimate the electrochemically active surface area (ECSA) of the catalyst, CV was tested by measuring *C*_dl_ between 1.10 to 1.30 V and between 0.0 to 0.2 V at various scan rates of 20–120 mV s^−1^ for OER and HER, respectively. The in situ Nyquist plots were measured with frequencies ranging from 100 kHz to 1 Hz, and the amplitude of 5 mV at a certain potential.

## Conflict of Interest

The authors declare no conflict of interest.

## Supporting information

Supporting information

## Data Availability

The data that support the findings of this study are available from the corresponding author upon reasonable request.
