# Supplementary material for: Bimetal Modulation Stabilizing a Metallic Heterostructure for Efficient Overall Water Splitting at Large Current Density
Source: Adv Sci (Weinh). 2022 Jul 11;9(25):2202750. doi: 10.1002/advs.202202750 (PMC9443435; doi:10.1002/advs.202202750)
Supplement: Supplementary file 1 — Supporting information [file ADVS-9-2202750-s001.pdf]

Supporting Information

Bimetal modulation stabilizing a metallic heterostructure for efficient overall water splitting at large current density

Tong Wu[#], Shumao Xu[#], Zhuang Zhang[#], Mengjia Luo, Ruiqi Wang, Yufeng Tang, Jiacheng Wang,* and Fuqiang Huang**

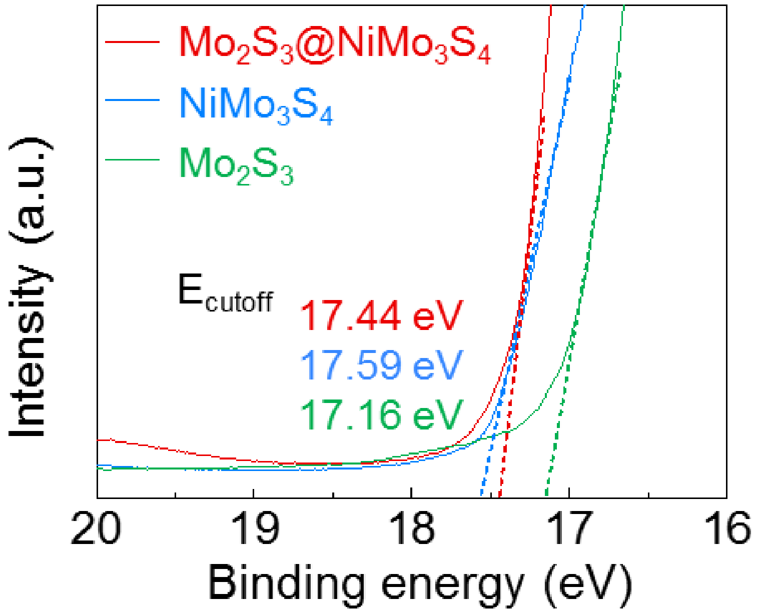

Figure S1. UPS spectra of secondary edge regions of Mo_2S_3@$NiMo_3S_4$, $NiMo_3S_4$, and Mo_2S_3.

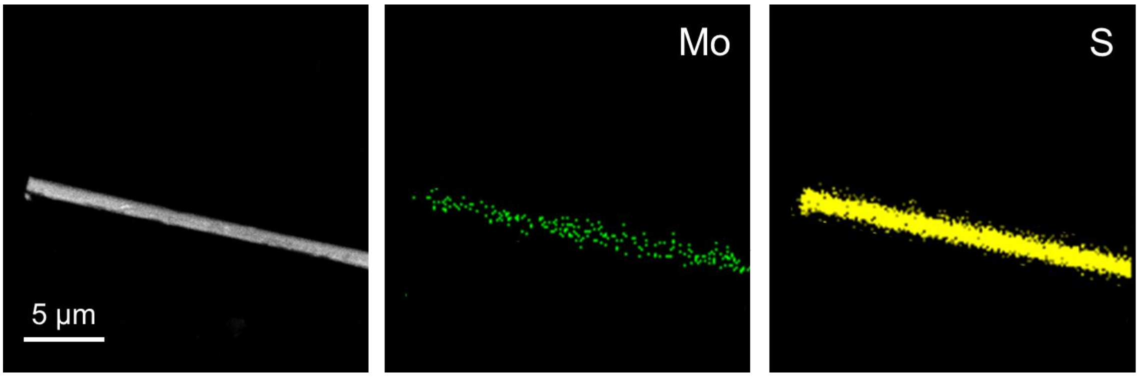

Figure S2. SEM and elemental mapping images of Mo_2S_3.

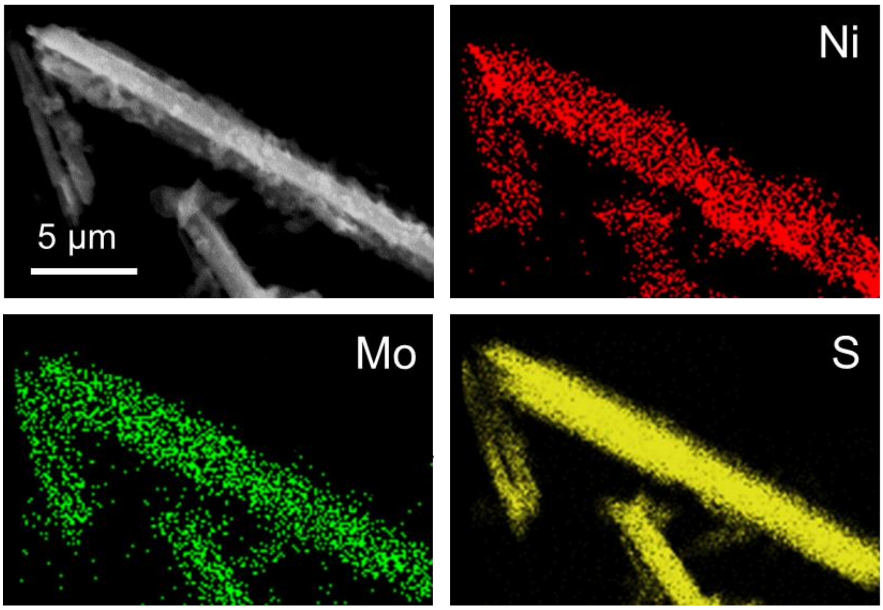

Figure S3. SEM and elemental mapping images of Mo_2S_3@$NiMo_3S_4$.

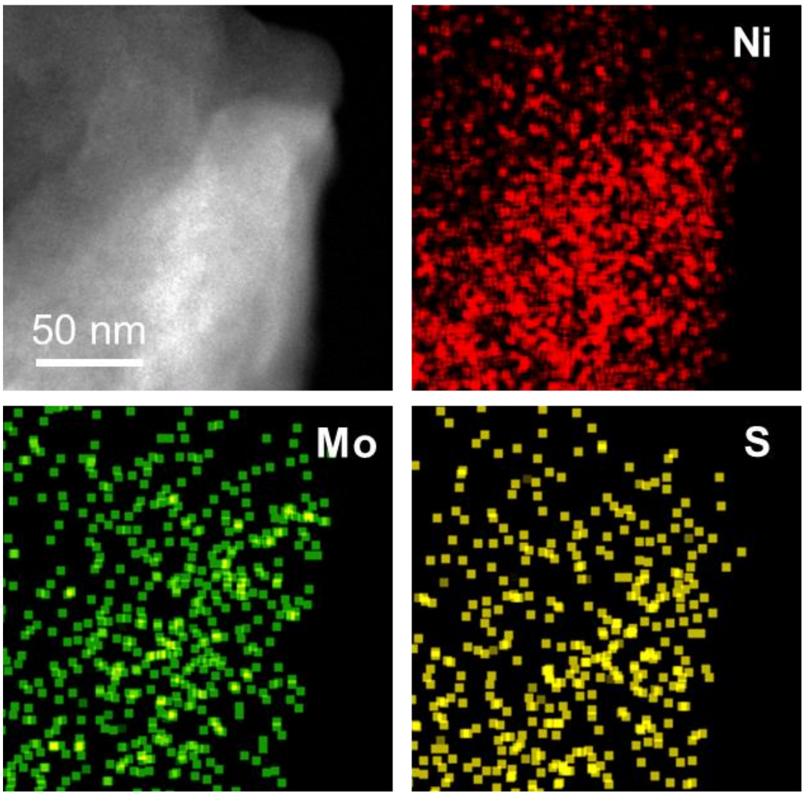

Figure S4. TEM and elemental mapping images of Mo_2S_3@$NiMo_3S_4$.

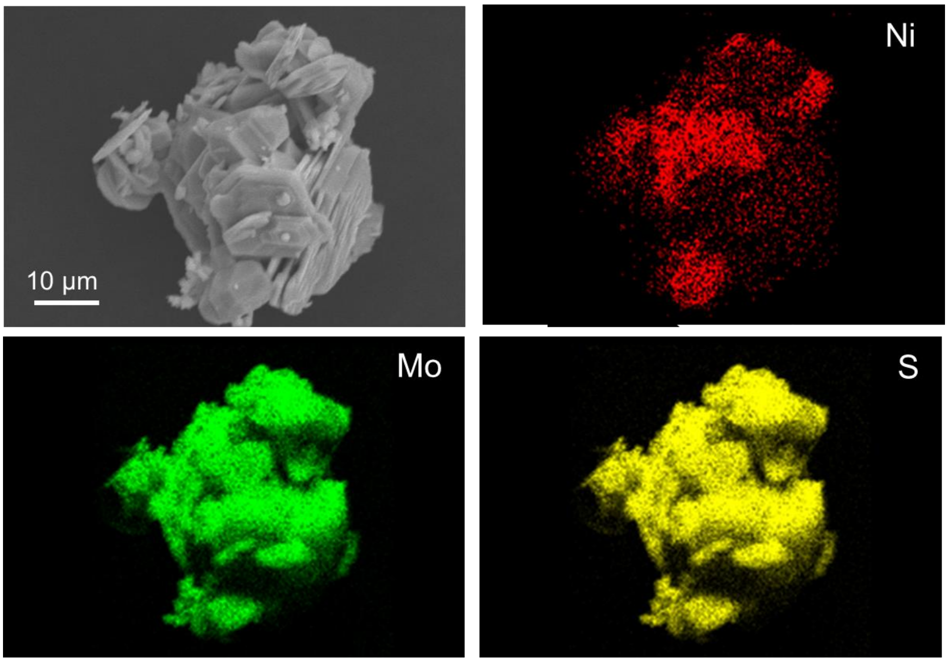

Figure S5. Typical SEM and corresponding elemental mapping images of $NiMo_3S_4$ nanosheets.

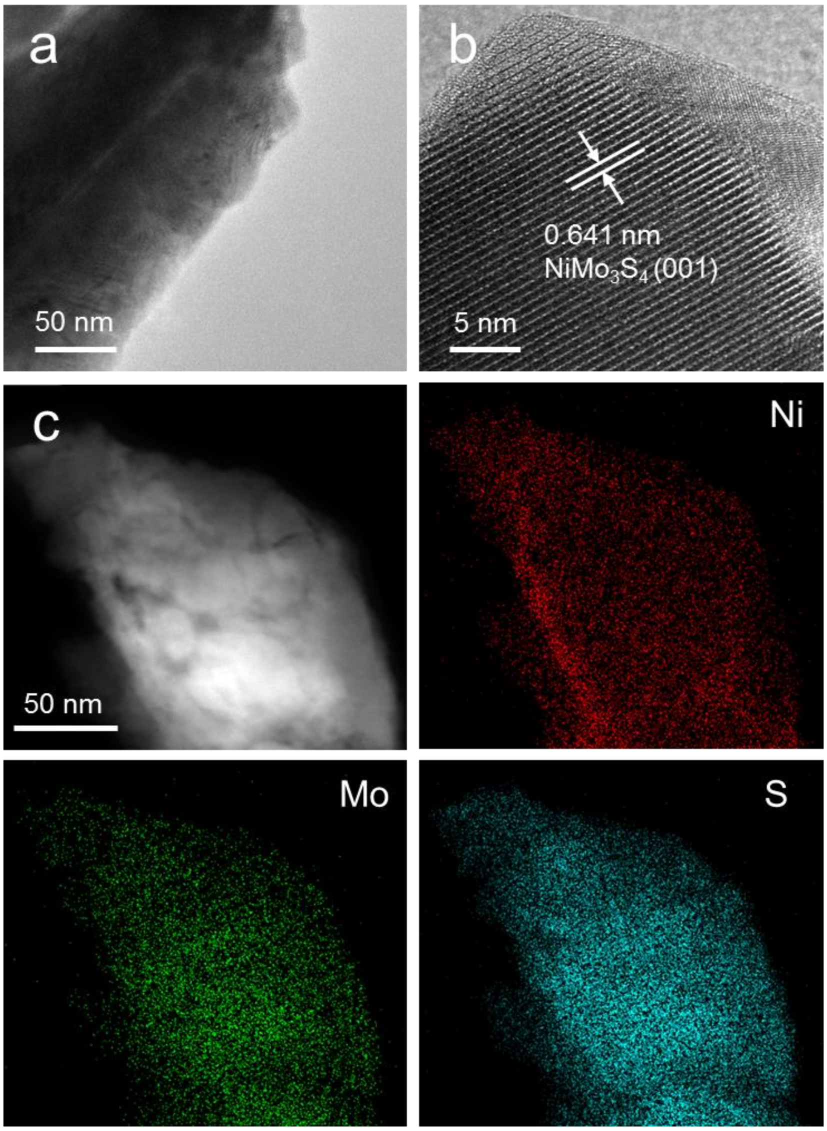

Figure S6. Morphologies of $NiMo_3S_4$ nanosheets. (a) Typical TEM image, (b) High-resolution TEM image, and (c) corresponding elemental mapping images of $NiMo_3S_4$ nanosheets.

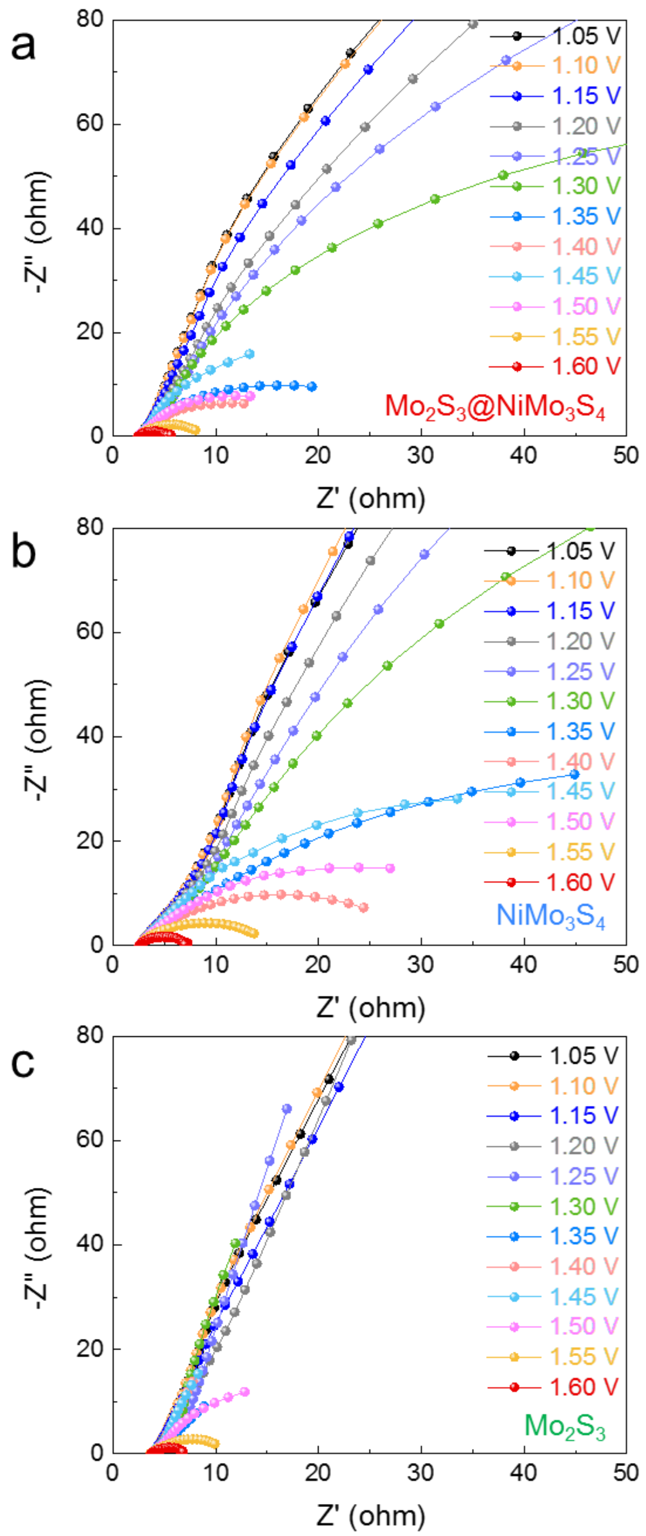

Figure S7. a-c) OER Kinetic analyses. *In-situ* Nyquist plots of (a) Mo_2S_3@$NiMo_3S_4$, (b) $NiMo_3S_4$, and (c) Mo_2S_3 at various potentials for OER, respectively.

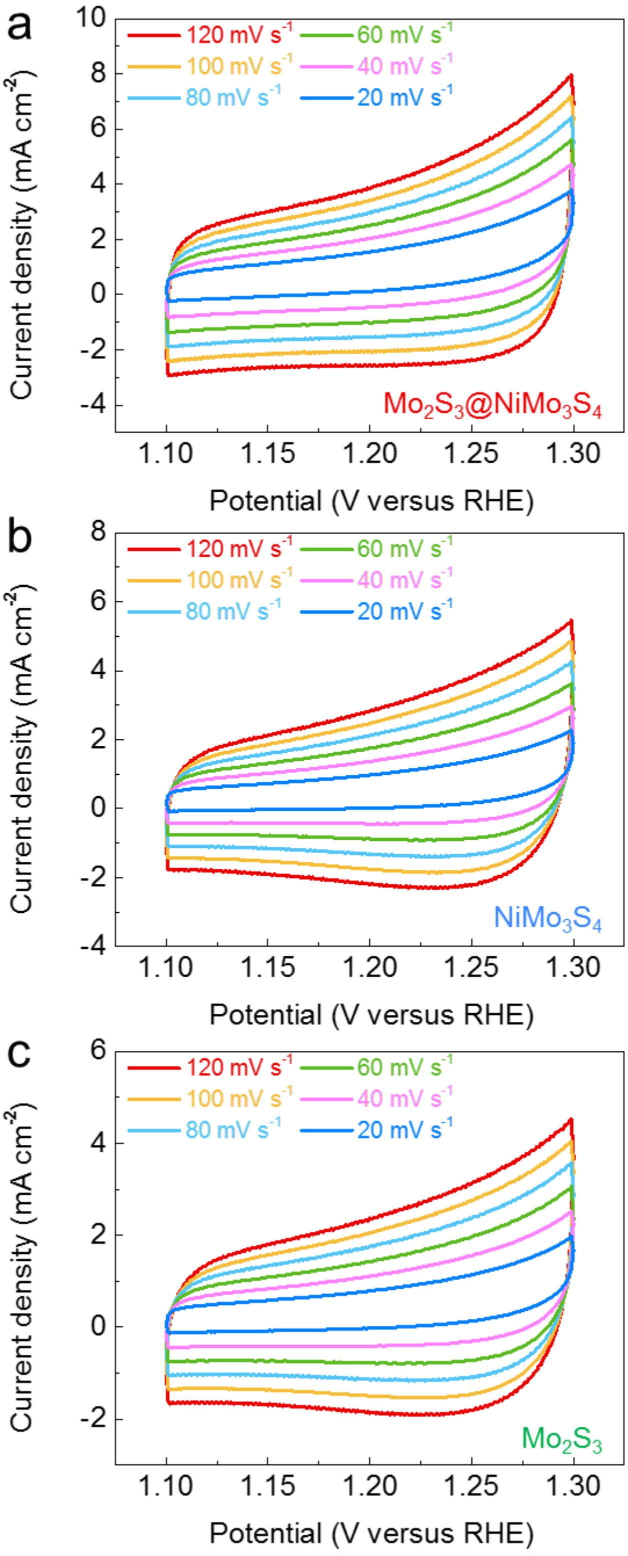

Figure S8. a-c) Cyclic voltammetry (CV) curves of (a) Mo_2S_3@$NiMo_3S_4$, (b) $NiMo_3S_4$, and (c) Mo_2S_3 measured at 1.10-1.30 V vs. RHE with different scan rates from 20 to 120 mV s^{-1}.

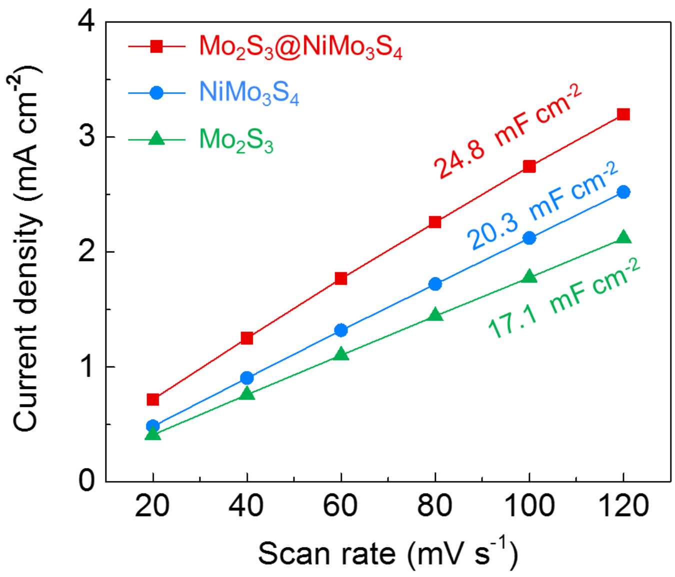

Figure S9. Active surface area. Plots were used to extract the double-layer capacitances (C_{dl}) and estimate the electrochemically active surface area of Mo_2S_3@$NiMo_3S_4$, $NiMo_3S_4$, and Mo_2S_3 for OER, respectively.

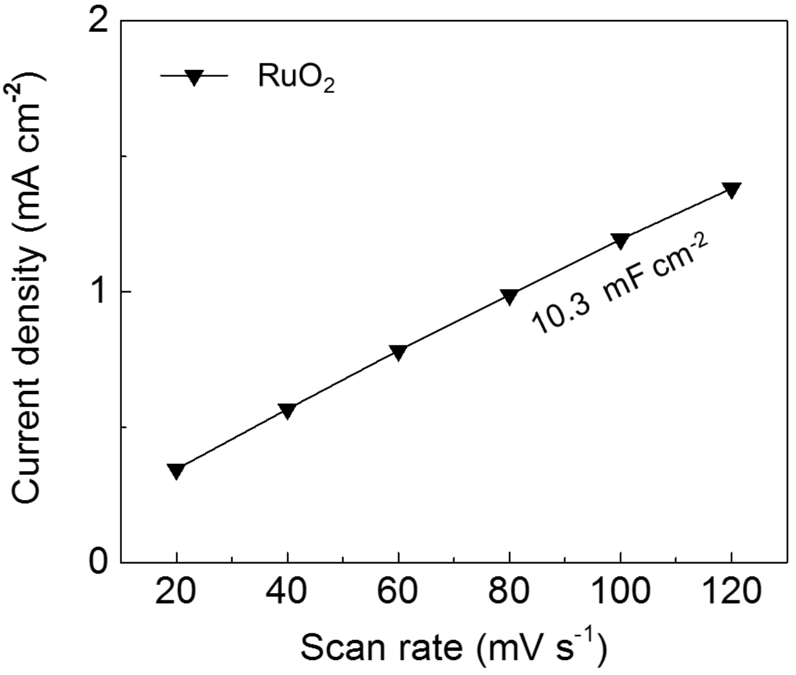

Figure S10. Current densities and scan rates plots to calculate the double-layer capacitances (C_{dl}) and estimate the electrochemically active surface area of commercial RuO_2 for OER.

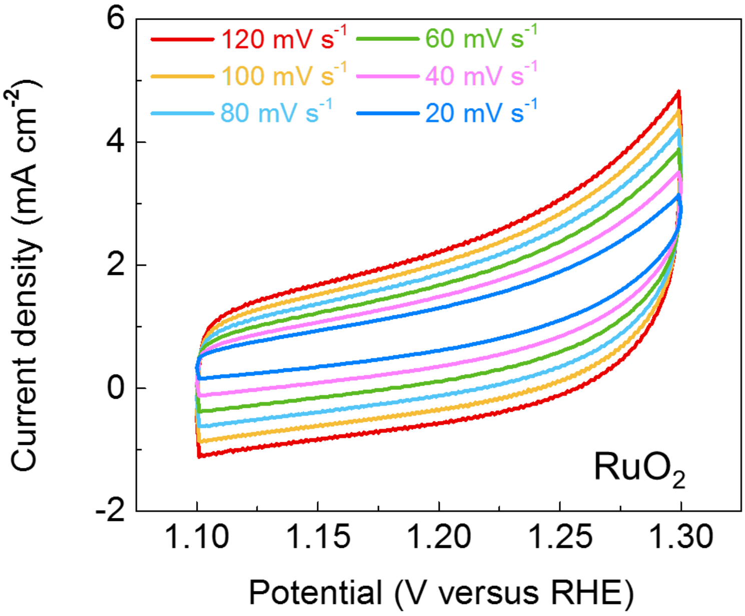

Figure S11. Cyclic voltammetry (CV) curves of commercial RuO_2 measured at 1.10-1.30 V vs. RHE with different scan rates from 20 to 120 mV s^{-1}.

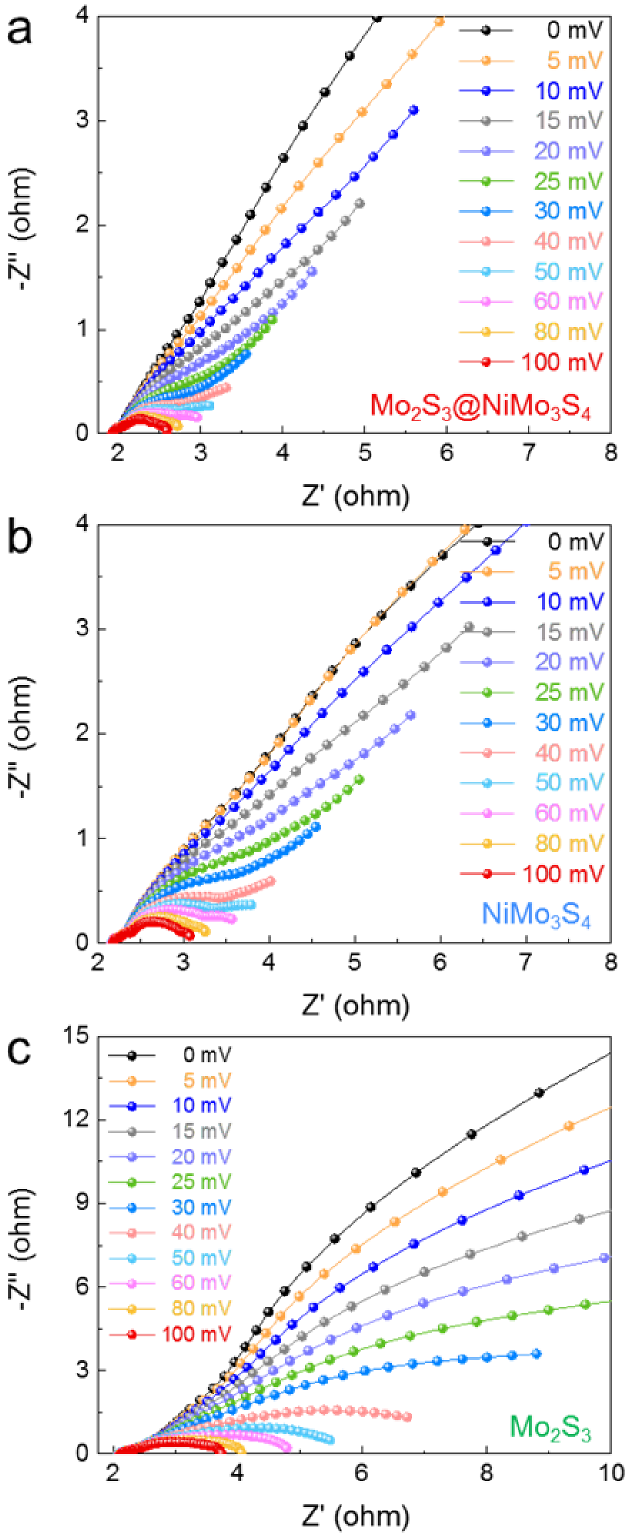

Figure S12. a-c) HER kinetic analyses. *in-situ* Nyquist plots of (a) Mo_2S_3@$NiMo_3S_4$, (b) $NiMo_3S_4$, and (c) Mo_2S_3 at various potentials for HER, respectively.

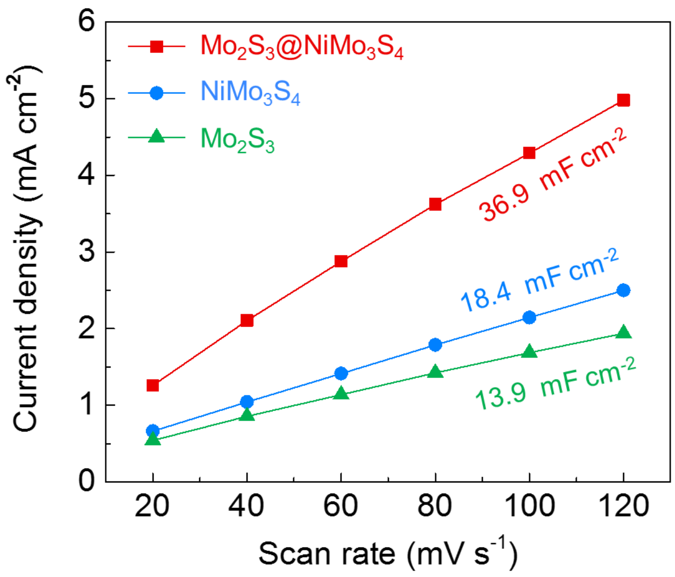

Figure S13. Current densities and scan rates plots to extract the double-layer capacitances (C_{dl}) and estimate the electrochemically active surface area of $Mo_2S_3@NiMo_3S_4$, $NiMo_3S_4$, and Mo_2S_3 for HER, respectively.

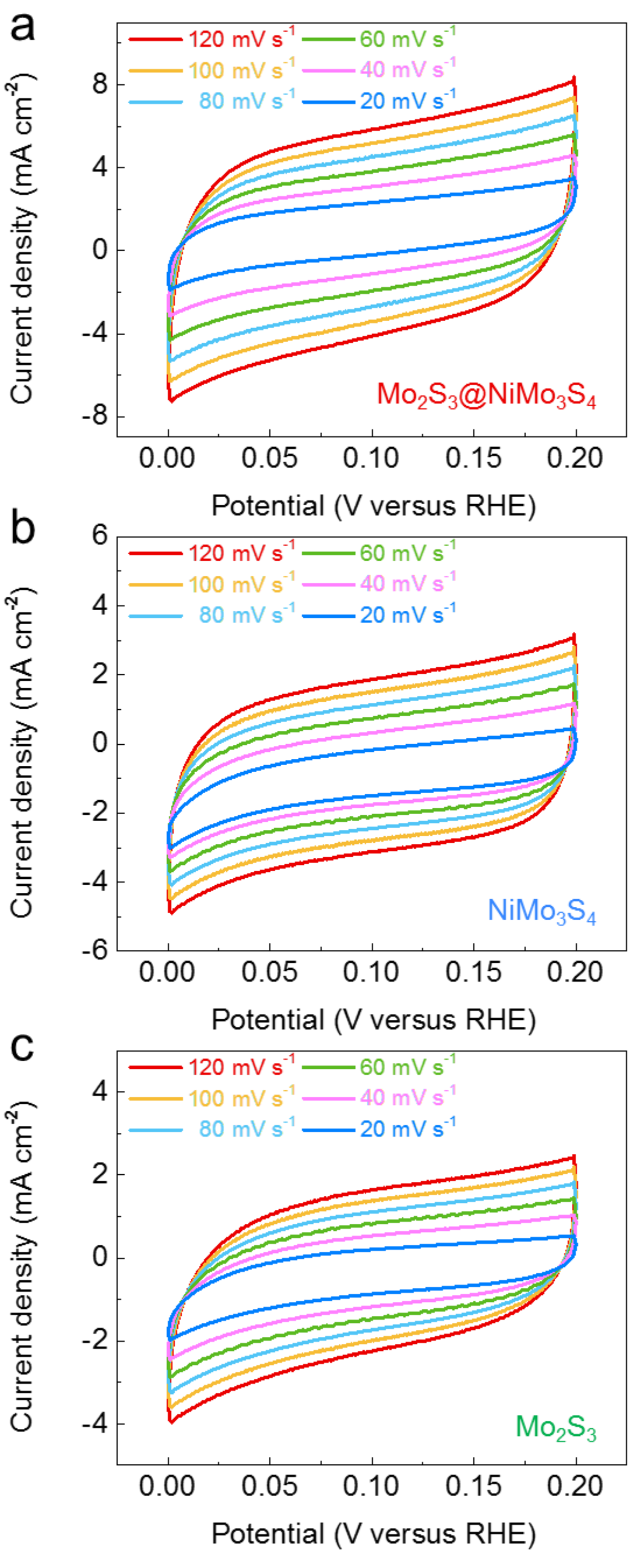

Figure S14. a-c) Cyclic voltammetry (CV) curves of (a) Mo_2S_3@$NiMo_3S_4$, (b) $NiMo_3S_4$, and (c) Mo_2S_3 measured at 0.00-0.20 V vs. RHE with different scan rates from 20 to 120 mV s^{-1}.

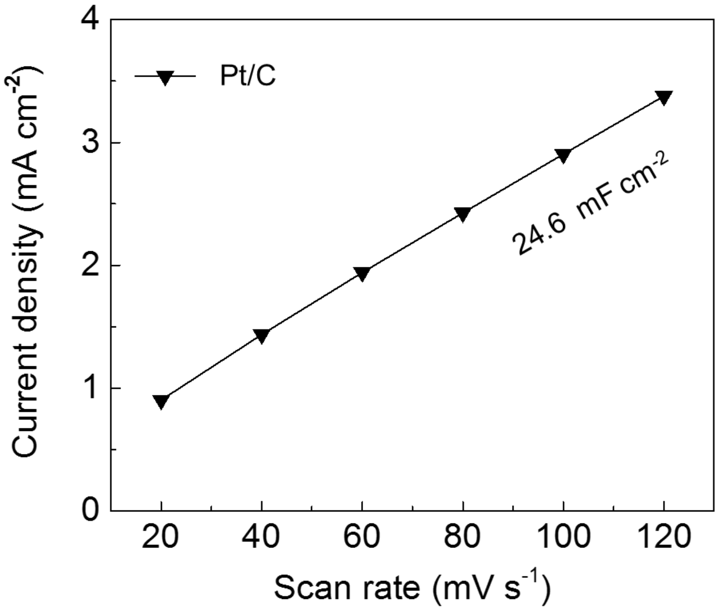

Figure S15. Current densities and scan rates plot to extract the double-layer capacitances (C_{dl}) and estimate the electrochemically active surface area of commercial Pt/C for HER.

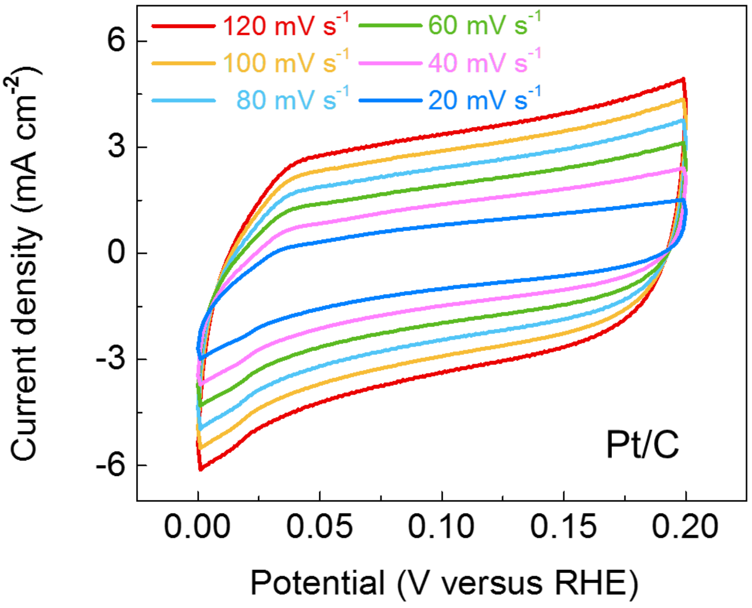

Figure S16. Cyclic voltammetry (CV) curves of commercial Pt/C measured at 0.00-0.20 V vs. RHE with different scan rates from 20 to 120 mV s^{-1}.

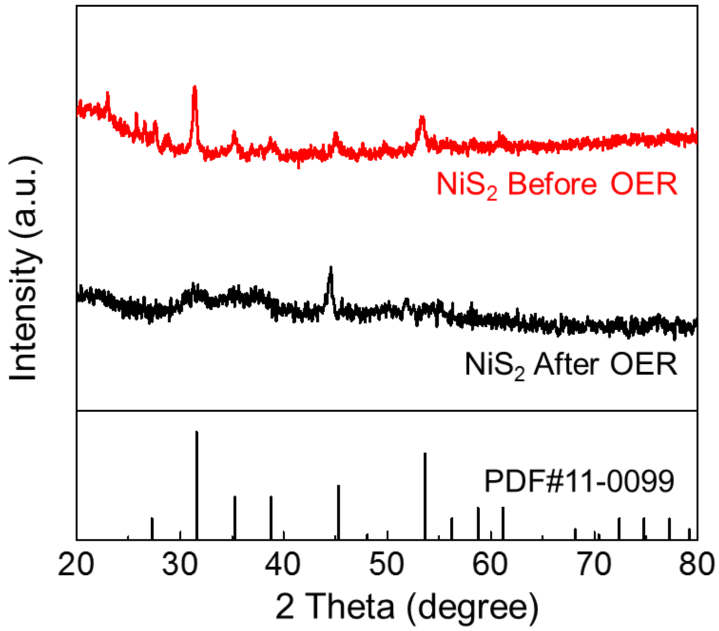

Figure S17. XRD patterns of NiS_2 before and after OER.

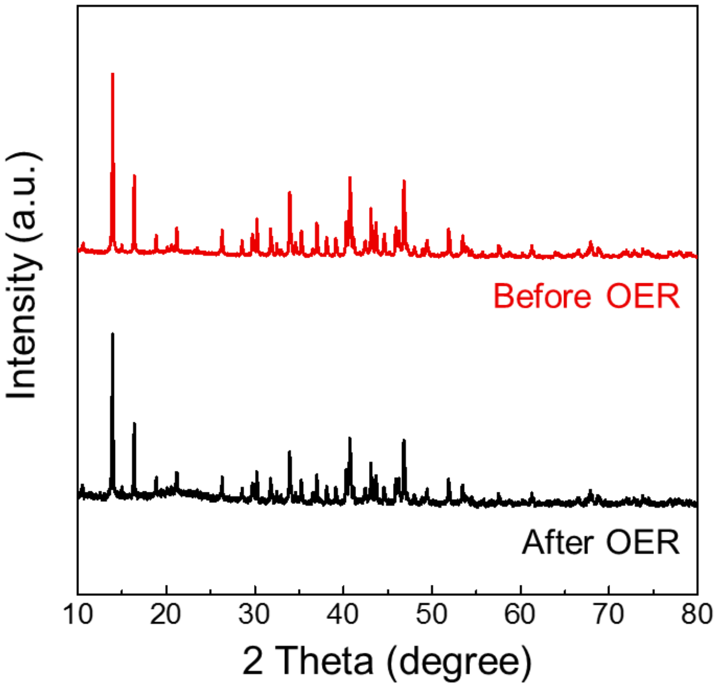

Figure S18. XRD patterns of Mo_2S_3@$NiMo_3S_4$ before and after OER.

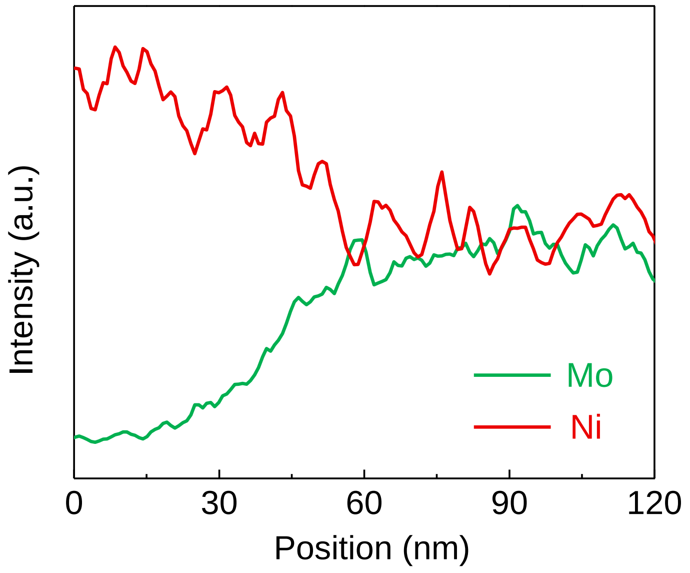

Figure S19. The energy dispersive spectroscopy (EDS) line scans of Mo and Ni elements for Mo_2S_3@$NiMo_3S_4$.

Table S1. Comparison of the OER activities of the Mo_2S_3@$NiMo_3S_4$ with the recently-reported catalysts.

**Catalysts**	**η_{10} (mV)**	**Tafel slope (mV dec^{-1})**	**Reference**
Mo_2S_3@$NiMo_3S_4$	**173**	**33.7**	**This work**
$Ni_{0.5}Co_{0.5}$-MOF-74	265	49	Nat. Energy 2020, 5, 881.
NiFeMo	180	/	Nat. Catal. 2020, 3, 985.
$Ni_{83}Fe_{17}$-ONCAs	190	39	Adv. Mater. 2021, 33, 2007377.
$Zn_{0.2}Co_{0.8}OOH$	235	35.7	Nat. Energy 2019, 4, 329.
Ru_1-Pt_3Cu	220	/	Nat. Catal. 2019, 2, 304.
$NiCo_{2-x}Fe_xO_4$ NBs	274	42	Angew. Chem. Int. Ed. 2021, 60, 11841.
Mn-NG	337	55	Nat. Catal. 2018, 1, 870.
$NiMoO_x$/NiMoS	201	47.2	Angew. Chem. Int. Ed. 2021, 60, 12328.
NiCo-UMOFNs	189	42	Nat. Energy 2016, 1, 1.

Table S2. Comparison of the HER activities of the Mo_2S_3@$NiMo_3S_4$ with the recently-reported catalysts.

Catalysts	η_{10} (mV)	Tafel slope (mV dec^{-1})	Reference
Mo_2S_3@$NiMo_3S_4$	**32**	**41.4**	**This work**
2H $Nb_{1.35}S_2$	40	43	Nat. Mater. 2019, 18, 1309.
L-Ag NPs	141	71	Nat. Catal. 2019, 2, 1107.
$CoMoS_x$/NF	89	94	Angew. Chem. Int. Ed. 2020, 59, 1659.
P-MoP/Mo_2N	89	78	Angew. Chem. Int. Ed. 2020, 133, 6747.
2H-MoS_2	190	50	Nat. Mater. 2016, 15, 1003.
NFP/C-3	95	72	Sci. Adv. 2019, 5, eaav6009.
SV-MoS_2	170	60	Nat. Mater. 2016, 15, 48.
MoO_2-FeP@C	103	48	Adv. Mater. 2020, 32, 2000455.
Cu NDs/Ni_3S_2 NTs-CFs	128	76.2	J. Am. Chem. Soc. 2018, 140, 610.

Table S3. Comparison of the overall water splitting performance of the $Mo_2S_3@NiMo_3S_4$ with the recently-reported catalysts at 1 A cm^{-2}.

Catalysts	OER (mV)	HER (mV)	Cell Voltage (V)	Reference
$Mo_2S_3@NiMo_3S_4$	**390**	**174**	**1.672**	**This work**
$MoNi_4$/SSW	/	161	2.1	Adv. Energy Mater. 2020, 10, 1904020.
$Ni_{0.8}Fe_{0.2}$-AHNAs	260	/	1.76	Energ. Environ. Sci. 2020, 13, 86.
NiMoN@NiFeN	398	218	1.841	Nat. Commun. 2019, 10, 5106.
Ni_2P-Fe_2P/NF	340	330	1.98	Adv. Funct. Mater. 2021, 31, 2006484.
Co_4N-CeO_2	491	280	2.3	Adv. Funct. Mater. 2020, 30, 1910596.
$NiMoO_x$/NiMoS	334	236	1.81	Nat. Commun. 2020, 11, 5462.
LiCoBPO	470	390	1.85	Energ. Environ. Sci. 2019, 12, 988.
2H-$Nb_{1.35}S_2$	/	270	2.0	Nat. Mater. 2019, 18(12): 1309.
FeP/Ni_2P	290	270	1.77	Nat. Commun. 2018, 9, 2551.
$CoMoS_x$/NF	442 (0.5 A cm^{-2})	269 (0.5 A cm^{-2})	2.04	Angew. Chem. Int. Ed. 2020, 59, 1659.
